# Method Paper – Distance and Travel Time to Casualty Clinics in Norway Based on Crowdsourced Postcode Coordinates: A Comparison with Other Methods

**DOI:** 10.1371/journal.pone.0089287

**Published:** 2014-02-14

**Authors:** Guttorm Raknes, Steinar Hunskaar

**Affiliations:** 1 National Centre for Emergency Primary Health Care, Uni Health, Uni Research, Bergen, Norway; 2 Department of Global Public Health and Primary Care, University of Bergen, Bergen, Norway; Universitat Rovira i Virgili, Spain

## Abstract

We describe a method that uses crowdsourced postcode coordinates and Google maps to estimate average distance and travel time for inhabitants of a municipality to a casualty clinic in Norway. The new method was compared with methods based on population centroids, median distance and town hall location, and we used it to examine how distance affects the utilisation of out-of-hours primary care services. At short distances our method showed good correlation with mean travel time and distance. The utilisation of out-of-hours services correlated with postcode based distances similar to previous research. The results show that our method is a reliable and useful tool for estimating average travel distances and travel times.

## Introduction

Travel distance is a major barrier to the utilisation of all kinds of health services. We have previously shown that increasing distance from the population centroid to a casualty clinic is strongly associated with decreasing contact and consultation rates in a Norwegian out-of-hours district. The association was almost completely independent from important demographic and socioeconomic factors registered [1].

Often no data on the exact location of individual patients are available. To examine the effect of geography on the utilisation of health services, several methods have been used to estimate the average travel distance for the entire population of an area. It has previously been shown that estimates of distance to the nearest cancer centre from population centroid is superior to estimates based on geometric centroid and population polygon methods when the exact addresses are unknown [2].

E.g. if a casualty clinic for a population of one municipality is located in another municipality, the distance from the population centroid is fairly representative for the average travel distance. In municipalities hosting their own casualty clinic, however, the population centroid often almost coincides with the location of the clinic. In these cases the distance between the population centroid and the casualty clinic is an underestimation of the travel distance for the average inhabitant.

In order to get more representative measures on distance and travel time at short distance between the casualty clinic and the population centroid of a municipality, we have developed a method that uses postcode coordinates that have been determined by crowdsourcing. The data are freely available for everyone, and since our method uses Google maps, there is no need for complicated and expensive geographic information system (GIS) software. In Norway, the alternative is to get estimates on population centroids or mean distances and travel times from Statistics Norway, the official statistics bureau. This is an expensive and time-consuming process.

Crowdsourcing is an online, distributed problem-solving and production model that involves outsourcing of tasks to a distributed group of people [3]. The contribution from thousands of voluntary people has been important for the development of geographic information systems (GIS) and related online resources [4].

In this paper we describe the method and compare it with other ways of calculating distance and travel time to a casualty clinic when the exact address is unknown. We also apply our method to examine whether distances and travel times are associated with the utilisation of out-of-hours primary care services.

## Methods

### Ethics Statements

The data used in this study are from three sources. Estimates on mean distance, travel time and population centroids of the municipality are not freely available, and can only be obtained from Statistics Norway for a fee. The postcode database is freely distributed under the terms of the Creative Commons Attribution License 3.0 [5], and is open to all from its website [6]. This database does only contain data on post/ZIP-codes. The Watchtower project database is owned and managed by our institution, the National Centre for Emergency Primary Health Care. The database is not publicly and freely available. These data are anonymised, patient identity is not recorded at any time.

The Watchtower project is approved by the Regional Committee for Medical and Health Research Ethics and by the Norwegian Social Science Data Services, the Data Protection Official for Research.

### Crowdsourcing Process

In 2009 a joint weather service [7] owned by the Norwegian Meteorological Institute and the Norwegian Broadcasting (NRK), wanted to provide weather forecasts for each of the 4 585 Norwegian postcodes. At that time, maps from Norway Post (Posten Norge) displaying the area covered by each postcode were available. Based on these data coordinates for the geometric centroids could be calculated. In a sparsely populated country like Norway, however, these coordinates are in many cases not representative for where people actually live.

To get more representative postcode coordinates, a crowdsourcing project was launched in July 2009. Volunteers were recruited using NRKbeta, the technology website of the Norwegian Broadcasting [8]. The head of the project, Erik Bolstad (EB), created a Google map with approximate locations of each postcode based on Norway Post’s downloadable postcode registry. Contributors were able to add more accurate coordinates directly by clicking in the map. Updated coordinates were received by Google forms and transferred to a Google spreadsheet. EB checked all submitted coordinates before updating the project webpage. Before the project started 39% of postcodes were located. After two days, this increased to 74%. Within the first month 4 590 feedbacks from approximately 600 persons were submitted. The capacity of the registration system was then exceeded due to the large amount of information. EB then had to find the location of the remaining 600 postcodes. A five point scale was used to assess the data quality of each location (Table 1). Since the formal termination of the project, the project pages have been open for volunteers to improve the database further. The database also contains data from Statistics Norway on the number of people belonging to each postcode [6].

**Table 1:** Quality of postcode based coordinates by a five point quality scale.

Status (scale value)	N
A. Actual post office location	1 633
B. Coordinate corrected by head of project	1 383
C. Coordinate controlled by a volunteer contributor	1 093
D. Coordinate corrected by a volunteer contributor	685
E. Probably misplaced	0

### Casualty Clinics

In Norway, casualty clinics (“legevakt”) are emergency primary care centres that handle all kinds of medical out-of-hours inquiries, including life-threatening incidents [9]. Some municipalities have their own casualty clinic (monomunicipal), but casualty clinics serving the population of two or more municipalities (intermunicipal) are also common. Of the 203 out-of-hours districts in Norway in 2012, 84 were monomunicipal and 88 were intermunicipal. The remaining districts were partially monomunicipal [10].

We used the postcode database to estimate average travel distance to the casualty clinic for 18 municipalities participating in the “Watchtower project”, a database of a Norwegian sentinel network for monitoring emergency primary health care activity in Norway. The database was established by the National Centre for Emergency Primary Health Care in 2007. The Watchtowers are seven out-of-hours districts that have been selected to be representative for the entire primary health care emergency service in Norway. The development and implementation of this database have been described in detail elsewhere [9].

### Calculation of Postcode Based Travel Time and Distance

The travel distance and travel time between each postcode coordinate within the Watchtower municipalities and the casualty clinic was calculated using Google maps [11]. This was then multiplied by the number of inhabitants belonging to the postcode. The average distance to the casualty clinic was then calculated by dividing the sum of person-kilometres or person-minutes by total number of inhabitants in the municipality. Instead of using GIS software to calculate distances and travel times, Google maps was chosen primarily because it is freely available to all. It is also easy for people without knowledge of GIS to calculate distances and travel times since the postcode database website has integrated Google maps functionality.

### Validation of the Method

The postcode based distances to casualty clinics for each municipality were compared with mean and median travel distances and travel times and distance from the population centroid of the municipality.

Mean distance and travel time of each municipality to the casualty clinic as calculated by Statistics Norway was considered the main basis of comparison for our method. Statistics Norway used Excel to manually calculate mean and median distance and travel time from all “address points” in the municipality, taking number of persons living at each address point into account. These data were taken from Statistics Norway’s Address Points database January 1^st^ 2012. This not freely available database contains the exact location of all addresses in Norway. Population centroids were calculated with the mean centre function in ArcInfo (ArcInfo, ESRI, Redlands, CA). Distances and travel times from population centroids were estimated using OD cost Matrix in ArcInfo. These estimates were commissioned from Statistics Norway for a fee. In addition, we made comparisons with distances and travel times between casualty clinic and the town halls of the municipalities, calculated by use of Google maps.

Comparisons of average absolute deviation from mean distance as calculated by Statistics Norway for each method were performed between mono- and intermunicipal out-of-hours districts, and between municipalities hosting a casualty clinic or not.

### Postcode Based Distance and Utilisation of Out-of-hours Services

We used the postcode based distances to examine the association between distance to casualty clinic and the utilisation of out-of-hours services. One municipality (Austevoll) was excluded from these analyses because the out-of-hours service uses more than one casualty clinic. Similar analyses on population centroid based distances have previously been performed by our group in the ten Watchtower municipalities comprising one specific out-of-hours district. These ten municipalities were among the 17 municipalities that were examined in the present study [1].

Utilisation was defined as rate of all first contacts with the communication centre of the out-of-hours district and rates of face-to-face doctor consultations in each of the Watchtower municipalities. Rates were calculated by dividing aggregated total number of contacts and consultations for each municipality in each year from 2007 to 2011 by population January 1st the same year.

### Statistical Methods

IBM SPSS 20 and Excel 2010 software were used to analyse data. The correlations between mean distance and travel time to casualty clinics and postcode based and other methods were examined using Pearson correlation coefficient. The correlation analyses were repeated several times, including municipalities at different maximum distances and travel times. Short distances were defined as less than ten kilometres, and short travel times defined as less than 15 minutes.

The magnitude of difference from mean distance calculated by Statistics Norway was expressed as mean absolute error (MAE) with 95% confidence intervals.

Contact and face-to-face consultations rates are number of events per year, which are assumed to be Poisson distributed. The rates of our main outcomes were so high that a normal distribution will fit the data well. Correlation coefficients, r, and constants and coefficients with 95% confidence intervals of exponential functions (non-linear regression) were used to assess any association between distances from municipality (postcode based) to out-of-hours-centre and contact and consultation rates for the years 2007–2011 for each municipality. Exponential curve fit was chosen because this was found to best describe the association between distance and utilisation of out-of-hours services in a previous study [1].

## Results

449 868 Watchtower contacts were registered from 2007 through 2011. 2 457 contacts were excluded because information about municipality was lacking. 135 770 contacts were excluded because the patient was a non-Norwegian citizen or was living in a municipality outside the Watchtowers out-of-hours districts. 311 641 contacts were eligible for analysis, aggregated to 90 municipality-year observations.

Baseline demographic and socioeconomic data for the municipalities are given in Table 2.

**Table 2:** Baseline data, Watchtower municipalities, sequence by mean distance to casualty clinic.

Municipality	Mean distance tocasualty clinic(kilometres)	Hosting acasualtyclinic?	Part ofintermunicipalout-of-hoursdistrict?	Population2011	Primarycare doctorsper10 000 (2011)	Population >80years (2011, %)
**Åsnes**	6.1	Yes	Yes	7 597	11.8	8.3
**Arendal**	6.3	Yes	Yes	42 229	5.6	4.5
**Alta**	6.6	Yes	No	19 071	11.9	2.7
**Nes**	7.6	Yes	No	19 049	6.6	4.0
**Tromsø**	8.8	Yes	No	68 239	10.4	2.7
**Kvam**	11.0	Yes	No	8 442	12.6	6.9
**Froland**	12.9	No	Yes	5 127	8.0	3.5
**Våler**	15.5	No	Yes	3 882	11.7	7.3
**Grimstad**	20.8	No	Yes	20 823	9.4	3.5
**Grue**	22.0	No	Yes	5 024	9.9	8.3
**Tvedestrand**	27.1	No	Yes	5 969	13.3	5.5
**Vegårshei**	38.5	No	Yes	1 922	8.8	5.9
**Risør**	47.5	No	Yes	6 871	9.6	6.0
**Gjerstad**	53.0	No	Yes	2 497	2.4	5.4
**Åmli**	53.1	No	Yes	1 829	19.2	5.5
**Nissedal**	96.3	No	Yes	1 405	18.2	6.1
**Fyresdal**	131.0	No	Yes	1 351	13.5	6.2

**Figure 1:**
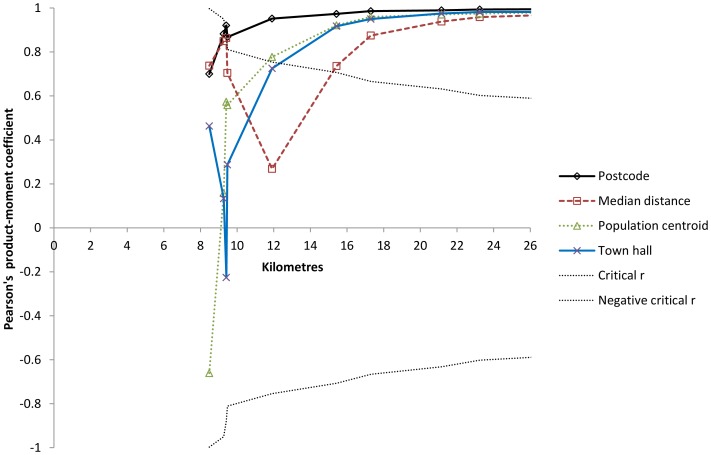
Correlation analysis of different distance measures at short distances by correlation coefficient, r, for the relationship between different average distance measures and mean travel time to casualty clinic. Analyses on municipalities with shorter travel times than indicated on the X-axis (N = 3 to 11). Critical r curves corresponding to p = 0.05. r-values between these curves are statistically non-significant.

Table 3 and Figure 1 display correlation analyses of the relationship of distance to the casualty clinic from different population centres and mean distance as computed by Statistics Norway. When analysing only municipalities with less than ten kilometres to a casualty clinic, only postcode based distances were significantly correlated with mean distances. Figure 1 shows the superior correlation of the postcode based method at distances less than 20 kilometres.

**Table 3:** Comparison of different distance measures to casualty clinic.

Distance (kilometres)	N	Postcode		Median		Population centroid		Town hall	
		r	p	r	p	r	p	r	p
**<135**	18	0.999	<0.001	0.999	<0.001	0.998	<0.001	0.997	<0.001
**<50**	14	0.999	<0.001	0.993	<0.001	0.992	<0.001	0.994	<0.001
**<30**	12	0.996	<0.001	0.974	<0.001	0.982	<0.001	0.983	<0.001
**<20**	9	0.986	<0.001	0.875	<0.001	0.960	<0.001	0.950	<0.001
**<10**	6	0.868	0.025	0.704	0.12	0.559	0.25	0.287	0.58

Correlation analysis of relationship between mean distance for inhabitants and other distance measures to casualty clinic. The analyses are performed for different maximum distances. r = correlation coefficient.

**Figure 2:**
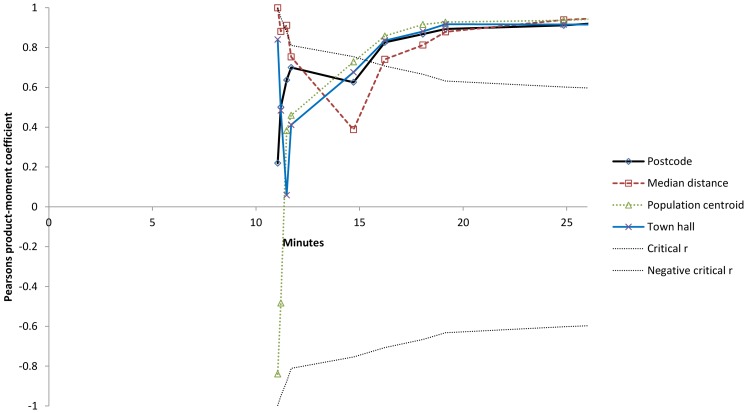
Correlation analysis of different travel time measures at short distances by correlation coefficients for the relationship between different travel time measures and mean travel time as calculated by Statistics Norway to casualty clinic. Analyses on municipalities with shorter distances than indicated on the X-axis (N = 3 to 11). Critical r curves corresponding to p = 0.05. r-values between these curves are statistically non-significant.

For travel time to casualty clinic, as shown in Table 4 and Figure 2, there were strong and statistically significant correlations for all time measures at long travel times. At travel times less than 15 minutes, none of the methods were significantly correlated with mean travel time as calculated by Statistics Norway. At longer travel times, the postcode based method showed a correlation to mean travel time similar to the other methods.

**Table 4:** Comparison of different travel time category measures to casualty clinic.

Time (minutes)	N	Postcode		Median		Population centroid		Town hall	
		r	p	r	p	r	p	r	p
**>120**	18	0.998	<0.001	0.999	<0.001	0.998	<0.001	0.995	<0.001
**<60**	16	0.994	<0.001	0.996	<0.001	0.992	<0.001	0.987	<0.001
**<45**	13	0.980	<0.001	0.986	<0.001	0.980	<0.001	0.960	<0.001
**<30**	11	0.911	0.004	0.939	0.002	0.937	0.002	0.916	0.004
**<15**	7	0.626	0.13	0.388	0.39	0.728	0.063	0.676	0.095

Correlation analysis of relationship between mean travel time for inhabitants and other travel time measures to casualty clinic. The analyses are performed for different maximum travel times. r = correlation coefficient.

Three of the municipalities had very similar mean distances and mean travel times that explain the abrupt change in critical r at 9.5 kilometres (Figure 1) and 11.5 minutes (Figure 2).

**Figure 3:**
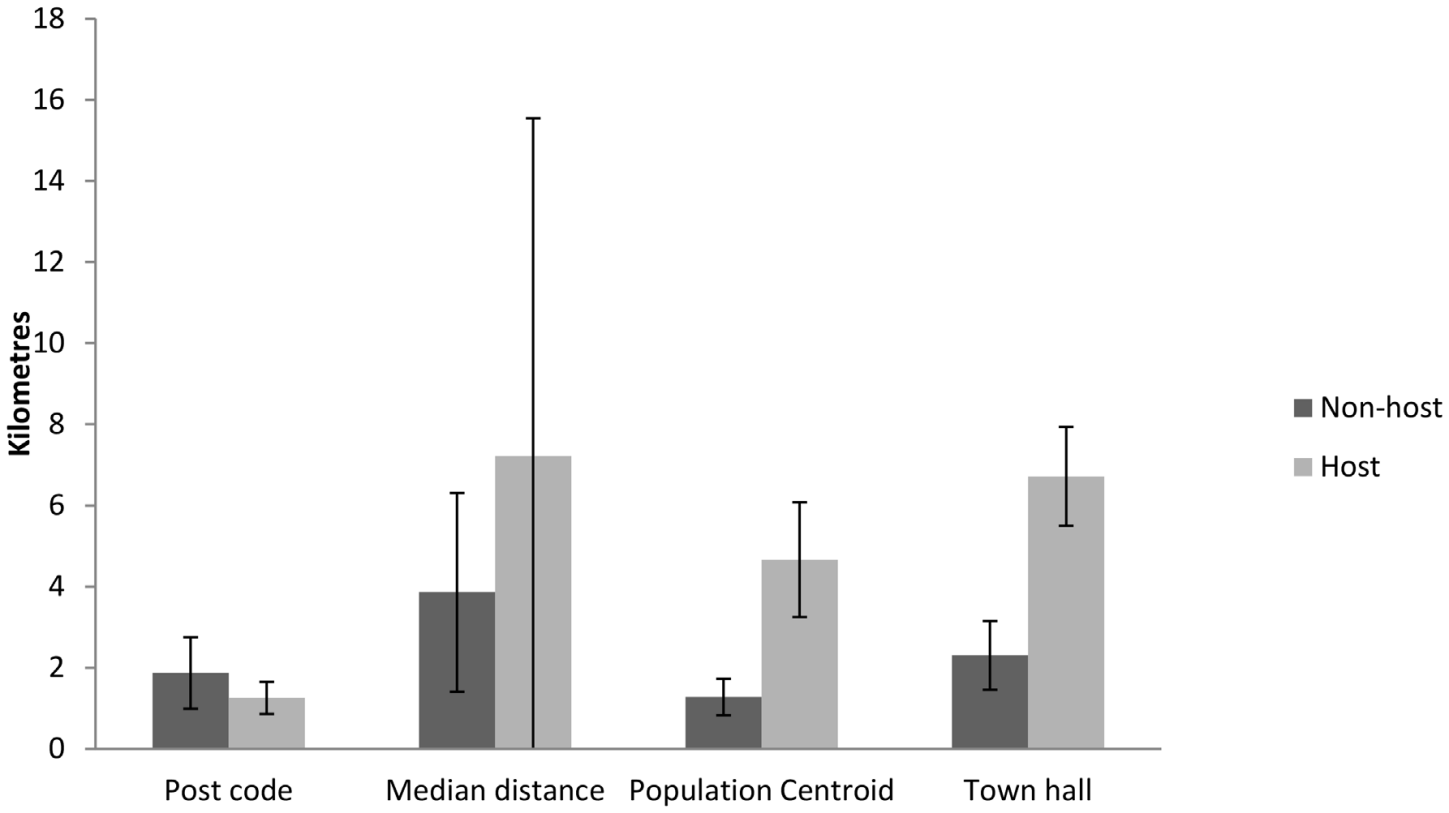
Mean absolute error (MAE) of distance measures in municipalities hosting a casualty clinic or not. Deviations (absolute) from mean distance to casualty clinic (kilometres) as calculated by Statistics Norway for the different distance measures. Error bars indicate 95% confidence intervals.

For municipalities not hosting a casualty clinic, there were no statistically significant differences in MAE between the distance measures compared with mean travel distance calculated by Statistics Norway (Figure 3). For distances based on population centroids and town halls, the deviation from mean distance was more than twofold larger in the municipalities where a casualty clinic is located. No statistically significant differences were observed in deviation between hosting and non-hosting municipalities in neither postcode nor median based distances, but the variance of deviation to mean distance was much higher for median based distances (figure 3).

**Figure 4:**
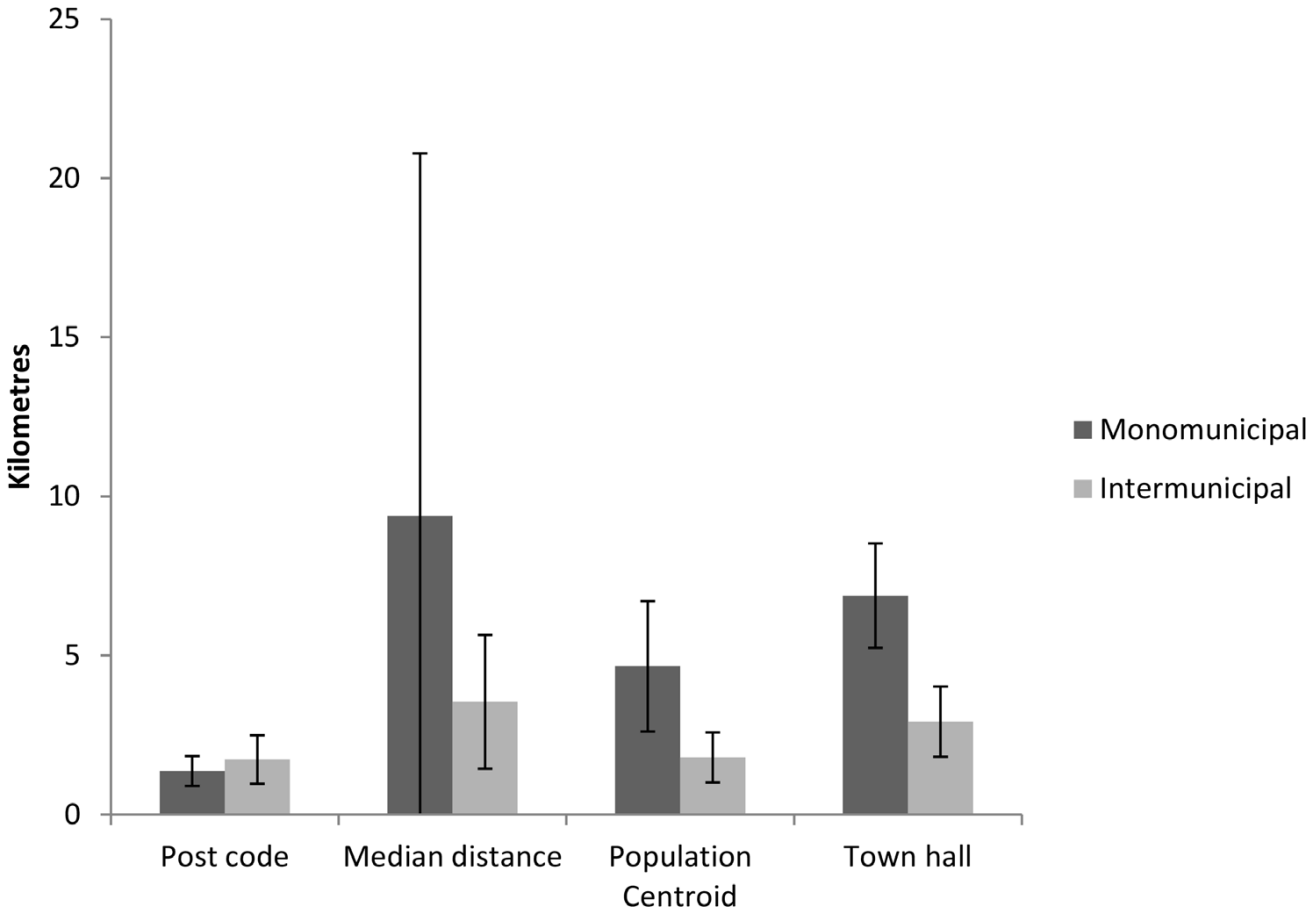
Mean absolute error (MAE) of distance measures in intermunicipal and monomunicipal out-of-hours districts. Deviations (absolute) from mean distance to casualty clinic (kilometres) as calculated by Statistics Norway for the different measures. Error bars indicate 95% confidence intervals.

For median and postcode based distances, there was no difference in MAE between mono- and intermunicipal out-of-hours districts. For distances based on population centroid and town hall location, the deviation from mean distance was more than twofold higher in municipalities not taking part in an intermunicipal out-of-hours district (Figure 4).

As shown in Tables 5 and 6, there were no significant differences in regression coefficients between different methods when analysing the association between distance and consultation and contact rates, but population centroid and town hall based distances differed more from mean distance, although not statistically significant.

**Table 5:** Distance and contact rates, nonlinear regression.

	Change0 to 50 km (%)	Constant	Coefficient	95% CI for coefficient	R^2^
**Postcode**	−45.5	464	−0.0121	−0.0133 to −0.0109	0.82
**Mean**	−45.0	470	−0.0120	−0.0132 to −0.0107	0.81
**Median**	−45.0	461	−0.0119	−0.0131 to −0.0107	0.81
**Population centroid**	−44.2	452	−0.0117	−0.0128 to −0.0105	0.82
**Town hall**	−43.8	444	−0.0115	−0.0126 to −0.0105	0.84

Relative change (%) in contact rates when moving 50 kilometres away from the casualty clinic for different distance measures. Constants and coefficients (with 95% confidence interval (CI)) for exponential functions.

**Table 6:** Distance and consultation rates, nonlinear regression.

	Change0 to 50 km (%)	Constant	Coefficient	95% CI for coefficient	R^2^
**Postcode**	−58.8	277	−0.01774	−0.0187 to −0.0168	0.94
**Mean**	−58.3	283	−0.01751	−0.0185 to −0.0165	0.93
**Median**	−58.2	275	−0.01745	−0.0184 to −0.0165	0.93
**Population centroid**	−57.4	267	−0.01706	−0.0179 to −0.0162	0.94
**Town hall**	−56.4	258	−0.01658	−0.0176 to −0.0156	0.93

Relative change (%) in consultation rates when moving 50 kilometres away from the casualty clinic for different distance measures. Constants and coefficients (with 95% confidence interval (CI)) for exponential functions.

**Figure 5:**
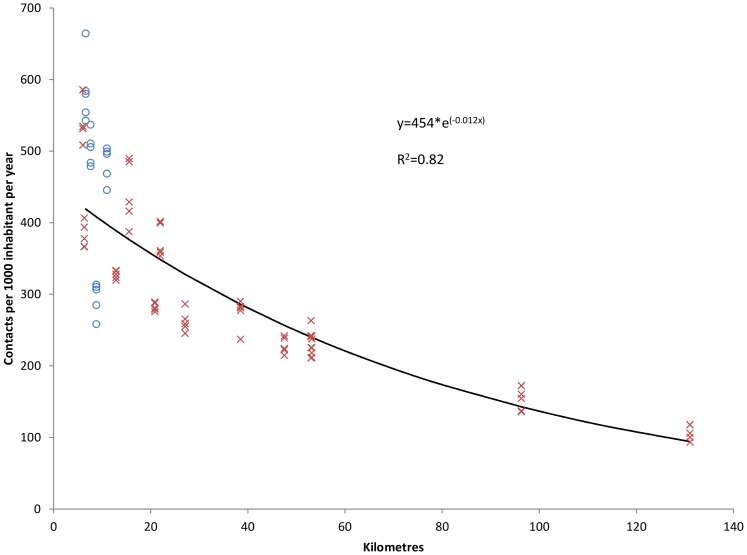
Postcode based distance and consultation rate at the casualty clinic. Relationship between average postcode based distance (kilometres) from Watchtower municipalities to casualty clinic and consultation rate at the casualty clinic (per 1 000 inhabitants per year). Each point represents rate in one municipality for one year (2007–2011). Circles indicate monomunicipal and crosses intermunicipal out-of-hours districts.

**Figure 6:**
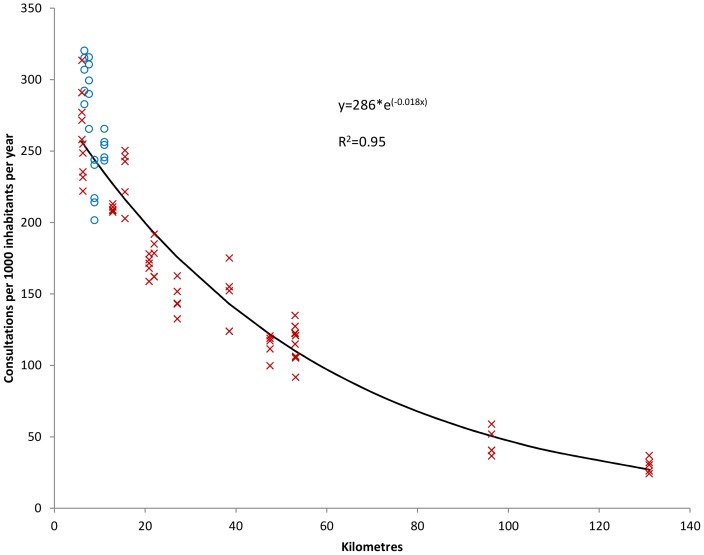
Postcode based travel time and consultation rate at the casualty clinic. Relationship between average postcode based travel time (minutes) from Watchtower municipalities to casualty clinic and consultation rate at the casualty clinic (per 1 000 inhabitants per year). Each point represents rate in one municipality for one year (2007–2011). Circles indicate monomunicipal and crosses intermunicipal out-of-hours districts.

Exponential regression showed that increasing (postcode based) distance was significantly associated with lower contact rate and to a larger extent face-to-face consultation rate (Figures 5 and 6). There was no difference between municipalities participating in intermunicipal or monomunicipal out-of-hours districts.

## Discussion

We have described a method for estimating distance and travel time to a casualty clinic based on postcodes determined by crowdsourcing. The method showed good correspondence to the main basis of comparison, which was mean distance and travel time based on address points. The method was at least as good as the other examined methods on both short and long distances. At distances shorter than 20 kilometres, the postcode based measure was superior to distances based on town hall location and population centroid. In contrast to distance and travel time measures based on town hall location and population centroid, the postcode based method showed no difference between mono- and intermunicipal out-of-hours districts, or between municipalities hosting a casualty clinic or not. The association between postcode based distance and contact and consultation rates in the 17 Watchtower municipalities was in accordance with our previous analysis of the 10 municipalities comprising Arendal out-of-hours district [1].

To calculate average travel distance and time from a municipality based on postcodes with the method described in this study, a database containing coordinates and population number for each postcode is necessary. The availability of such data is for most countries more limited than in Norway. There are some on-line services in UK that provide coordinates of postcodes [12], [13], but to our knowledge information on the population of each postcode is not readily available. Some commercial providers, like Deutsche Post in Germany provide data based on addresses. Another limitation is that we used Google maps to calculate road distances and travel times. The reliability of Google Maps presumably varies from area to area because map data are acquired from different sources. Crowdsourcing projects with large numbers of contributors result in products that are complete, accurate and up to date, but they are vulnerable to errors and vandalism. Research on Wikipedia has shown that strict coordination of the editing process is crucial for quality [14]. The thorough planning and coordination of the more than 600 contributors of the postcode project suggests that the database is trustworthy.

### Conclusion

The results show that distances and travel times for the inhabitants of Norwegian municipalities to a casualty clinic can be easily calculated with standard Internet tools from freely available postcode based coordinates determined by crowdsourcing. The method described here proved valid at both long and short distances, and is more reliable than distance estimates based on population centroid at short distances.
